# Diet-regulated transcriptional plasticity of plant parasites in plant–mutualist environments

**DOI:** 10.1073/pnas.2421367122

**Published:** 2025-04-17

**Authors:** M. Willow H. Maxwell, Barry E. Causier, Jasper Chippendale, James R. Ault, Chris A. Bell

**Affiliations:** ^a^School of Biology, Faculty of Biological Sciences, University of Leeds, Leeds LS2 9JT, United Kingdom; ^b^School of Molecular and Cellular Biology, Faculty of Biological Sciences, University of Leeds, Leeds LS2 9JT, United Kingdom

**Keywords:** symbionts, parasite, mutualist, plant-parasitic nematodes, plant resources

## Abstract

Crop pathogens often lack exclusive access to their host and must interact with plants concurrently engaged with numerous other symbionts. Here, we demonstrate that the colonization of hosts by plant–mutualistic mycorrhizal fungi can indirectly induce transcriptional responses of a major plant parasite, the nematode *Globodera pallida*, via a modified host resource profile. A shift in the resource profile of the root, where the parasite feeds, is perceived and responded to by the parasite through transcriptional changes, potentially to optimize resource intake. Specifically, *G. pallida* react to reduced host-photosynthate influx due to concurrent mycorrhizal-host symbiosis by upregulating the expression of a sugar transporter (*SWEET3*) in the nematode intestine. We identify this gene’s role in parasite growth and development, regulated by the putative diet-responsive transcription factor Gp-HBL1. Overall, our data unveil a mechanism by which a parasitic animal responds to fluctuations in host plant quality that is induced by a plant–mutualistic fungus, to enhance parasitism and reproduction.

## Significance

Successful parasitism, regardless of the taxonomy of the host, relies upon the acquisition of host resources by the parasite. As well as with parasites, plants interact with a myriad of organisms within soils, including beneficial mutualists. Here, we outline the ability of a parasite to transcriptionally respond to fluctuations in food intake that are induced by a plant-beneficial fungus competing for the finite reserve of host resources. This adaptive mechanism potentially supports the long-term biotrophy of pathogens in diverse and dynamic living conditions and “off-sets” deficiencies that may be induced by interkingdom competition.

Obligate biotrophic pathogens must acquire food from their hosts in order to survive and reproduce. “Feeding” is therefore central to the infection cycle, whether the pathogen is bacterial, fungal, or animal. Preventing pathogen feeding is an attractive aim of control strategies, as a reduction in feeding often relates to a reduction in growth and a reduced pathogen population.

The importance of timely, regulated feeding by the pathogen is exemplified by the cyst nematode, *Globodera pallida*, which can cause yield losses of up to 80% of potato crops (1). This devastating global parasite of potato plants may persist in soils for decades as dormant eggs (2) until a host plant is detected, at which point the infective stage nematode hatches and invades the nearby root system, utilizing only its innate lipid stores. If the nematode is able to migrate to the vascular tissue and establish a successful feeding site, then it will be able to fuel the rest of its life cycle, ultimately producing a second generation. Cyst nematode nutrition is acquired from a specialized feeding structure induced within the root, which has elevated metabolic activity and acts as a “nutrient sink” for plant resources (3). This structure, termed a syncytium, is created by the fusion of up to hundreds of root cells. The nematode relies on this single syncytium for the entirety of its nutrition, which sustains development from juvenile to adult—between 4 and 6 wk in total (4). If nematode feeding is prevented (5), or the food source (i.e. plant) is of lesser “quality” [e.g., large density of competing nematodes (6) or reduced nutritional status (7)], then there will be a deleterious impact on female nematode growth and the number of eggs produced. Although plant-parasitic nematodes are known to produce a range of enzymes that facilitate the breakdown of host compounds within the nematode gut [e.g., endoglucanases (8), proteinases (9–11), and detoxifying enzymes (12)], little is known about how the products of digestion are absorbed from the intestine and utilized by the nematode.

Concurrently colonizing biotrophic symbionts compete for resources and nutrients from the host plant (13), but how this might affect the regulation of pathogen feeding has not previously been explored. Cyst nematodes provide an excellent study system as their prolonged feeding period compared to other plant pathogens will coincide with, and potentially outlast, other plant–symbiont interactions, resulting in a dynamic influx of resources to the parasite. Arbuscular mycorrhizal (AM) fungi are obligate biotrophs that have the potential to improve crop performance (e.g., ref. 14), however they concurrently cause an increase in egg production of *G. pallida* when cocolonizing potato plants with the pathogen (15). This effect on reproduction may be mediated via changes to the nutrition of the nematode. For example, *G. pallida* feeding from AM fungal-colonized potato roots contained elevated concentrations of phosphorus and nitrogen yet reduced concentrations of plant-fixed carbon, compared to nematodes feeding on non-AM fungal-colonized hosts (15, 16). This implies that reduced carbon in nematode food intake, at the scale observed, has less impact on nematode fitness than other components of the diet. It is possible that the nematode can detect varying quality of food intake and respond in such a way as to ameliorate, within limits, any negative effects, but if and how that occurs during concurrent plant–mutualist interactions is currently unknown.

Sugars will eventually be exported transporters (SWEETs) are a family of sugar efflux transporters that are widespread in eukaryote genomes (e.g., a single human copy, 17 genes in *Arabidopsis thaliana*, and 7 in *Caenorhabditis elegans*) (17). Plant SWEETs are an increasingly well-researched family of genes with Clade I/II SWEETs suggested to solely transport glucose whereas Clade III transport sucrose (17) and Clade IV translocate fructose (18). As well as playing major roles in plant growth and development, SWEETs directly facilitate the movement of hexoses, predominantly glucose, into the feeding site of the plant-parasitic nematode *Meloidogyne incognita*, therefore contributing toward host susceptibility to these pests (19, 20). Other plant pathogens also induce expression of host SWEET genes to promote the movement of hexoses toward the pathogen, indicating them to be general susceptibility genes (17, 21, 22). Although SWEET characterization in plant disease resistance is expanding, their identification and characterization in the plant parasites and pathogens themselves is underexplored (23, 24). The mechanisms underpinning the utilization and absorption of plant sugars by the parasite is unknown, although similar hexose transporters within nematodes are highly likely to play a role.

Here, we present data to suggest that while plant–mutualist interactions may positively impact host nutrition, these changes are perceived by and induce transcriptional responses in plant parasites to maximize their food intake and reproduction. This display of transcriptional plasticity may be a mechanism to support long-term parasitism of hosts and may be conserved in prolonged parasitic lifestyles.

## Results

**Fig. 1.**
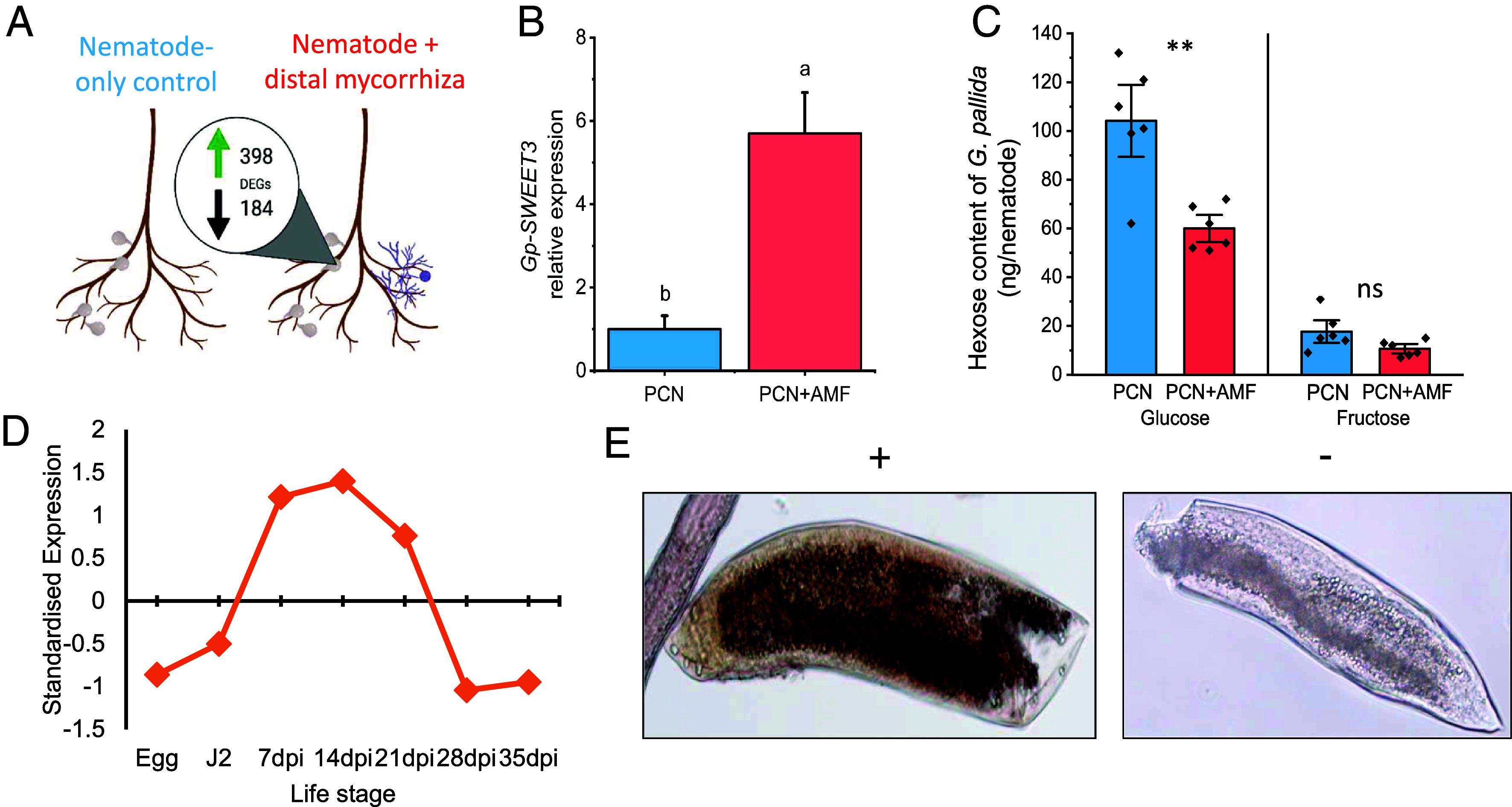
Identification and characterization of the potentially diet-responsive *Gp-SWEET3.* The impact of distal colonization of potato roots by AM fungi on coinfecting *G. pallida.* (*A*) The gene expression profile of *G. pallida* from root systems ± AM fungi (*P* < 0.001; FDR DESeq2). (*B*) *Gp-SWEET3* expression of *G. pallida* from root systems ± AM fungi (*P* < 0.001; FDR DESeq2). (*C*) Glucose and fructose content of *G. pallida* from root systems ± AM fungi (** denotes significance at *P* < 0.01, ns indicates *P* = 0.056; Two-sample *t* test). (*D*) Temporal expression of *Gp-SWEET3* throughout the *G. pallida* life cycle (25). (*E*) Spatial expression of *Gp-SWEET3* via in situ hybridization with digoxigenin probes on parasitic life stages (+ antisense probe, − negative control sense probe). Four biological replicates were conducted for *A*–*C*.

Host–AM fungi interactions lead to a reduction in plant-carbon-based compounds ingested by concurrent, competing *G. pallida* (15). We compared the gene expression profile of *G. pallida* infecting potato root systems that were distally colonized by AM fungi with that of nematodes on root systems absent of AM fungi, to understand the effects of this variation in diet (Fig. 1*A*). A split-root setup was used to ensure no direct interactions between nematodes and fungi and that any observed effects were mediated through the host. Alignment of RNAseq reads from infected potato root tissue to a *G. pallida* genome assembly (*SI Appendix*, Table S1) followed by differential expression analysis suggested that colonization by AM fungi and associated changes in systemic host resource profiles (15, 16) led to altered gene expression of feeding nematodes. AM fungal colonization resulted in the up-regulation of 398 genes and down-regulated of 184 genes in *G. pallida* infecting the same host, compared to those from AM-free root systems (Fig. 1*A*; FDR < 0.001, 1 < log2 fold change < −1; *SI Appendix*, *Supplementary File* 1). Further, 147 out of the 184 *G. pallida* genes that were down-regulated on AM-colonized hosts had no predicted annotation or related GO term. Of the 37 annotated genes, there was an enrichment in functions related to lipid/fatty acid/protein modifications, thiol oxidation, and nematode development. A large array of functions were represented among the 398 up-regulated *G. pallida* genes, notably resource transport/digestion (lipid/fatty acid modifications, carbohydrate transport), effectors, cuticle/collagen modifications, and neuropeptide-signaling (*SI Appendix*, *Supplementary File* 1).

Genes with functions related to resource movement and digestion were filtered from the dataset due to the known impact of concurrent AM-colonization on *G. pallida* resource intake (15). This identified *Gp-SWEET3*, a transmembrane hexose transporter, as up-regulated 5.4-fold in *G. pallida* from AM-colonized hosts (Fig. 1*B*; DESeq2; FDR < 0.001). We then quantified the predicted substrates of *Gp-SWEET3* in nematodes from AM-colonized potato roots to determine the link between differential gene expression and substrate flow. The predicted substrates for SWEET3, glucose and fructose, were found to be reduced in *G. pallida* that were parasitizing AM-colonized hosts, compared to nematode-only plants (Fig. 1*C*). This suggests that *Gp-SWEET3* was upregulated when the substrate quantity was reduced.

*Gp-SWEET3* is expressed during feeding stages of *G. pallida* (Fig. 1*D*; notably 7 to 21 dpi; (25) and within the intestine of the nematode (Fig. 1*E* and *SI Appendix*, Fig. S2). All of the available plant-parasitic nematode genomes contain orthologs of *C. elegans* SWEET genes, indicating their wide conservation (*SI Appendix*, Fig. S3). Although the number of SWEET genes varied between the plant-parasitic nematode species (*SI Appendix*, Table S2), *SWEET3* was present in all species including *Pratylenchus coffeae*, which only has two SWEET genes and the smallest reported plant-parasitic nematode genome (26). SWEET phylogeny generally mirrored the typical 18S phylogeny (27), with additional clustering related to the expression profile of each SWEET throughout the nematode’s life cycle. Specifically, *SWEET 3, 5, 6*, and *7* are expressed most highly during feeding stages of *G. pallida, Heterodera schachtii,* and *M. incognita*, while *SWEET 1, 2,* and *4* have the highest expression during mobile life stages of these nematodes (*SI Appendix*, Fig. S3).

RNA interference (RNAi) was conducted to further investigate the role of *Gp-SWEET3* in feeding and nematode development. Expression of *Gp-SWEET3* was reduced after the incubation of *G. pallida* J2s in *Gp-SWEET3*-targeting small RNA (siRNA) for 16 h (*SI Appendix*, Fig. S4). The greatest reductions in gene expression were recorded 24 h after incubation, with knockdown effects present but diminishing at 7 d after exposure. All three independent siRNAs reduced expression *of Gp-SWEET*3 in J2s up to 10 d postexposure (−28%, −37%, and −16%) and a combined treatment offered no additive effect but still a consistent knockdown (−32%).

Once the efficacy of RNAi in J2 nematodes was confirmed, fresh batches of siRNA-treated *G. pallida* J2s were inoculated onto potato roots. After 10 d, parasitizing nematodes treated with siRNA (either three siRNAs independently or combined) showed significant reductions in *Gp-SWEET3* expression (*SI Appendix*, Fig. S5*A*; grand mean 27% reduction relative to control; *P* < 0.05; One way-ANOVA, SNK). *Gp-SWEET3* knockdown did not result in a reduction in nematode invasion (*SI Appendix*, Fig. S5*B*) but did reduce the surface area of invaded nematodes (*SI Appendix*, Fig. S5*C*; grand mean of −42% compared to control; *P* < 0.01; One way-ANOVA, SNK).

**Fig. 2.**
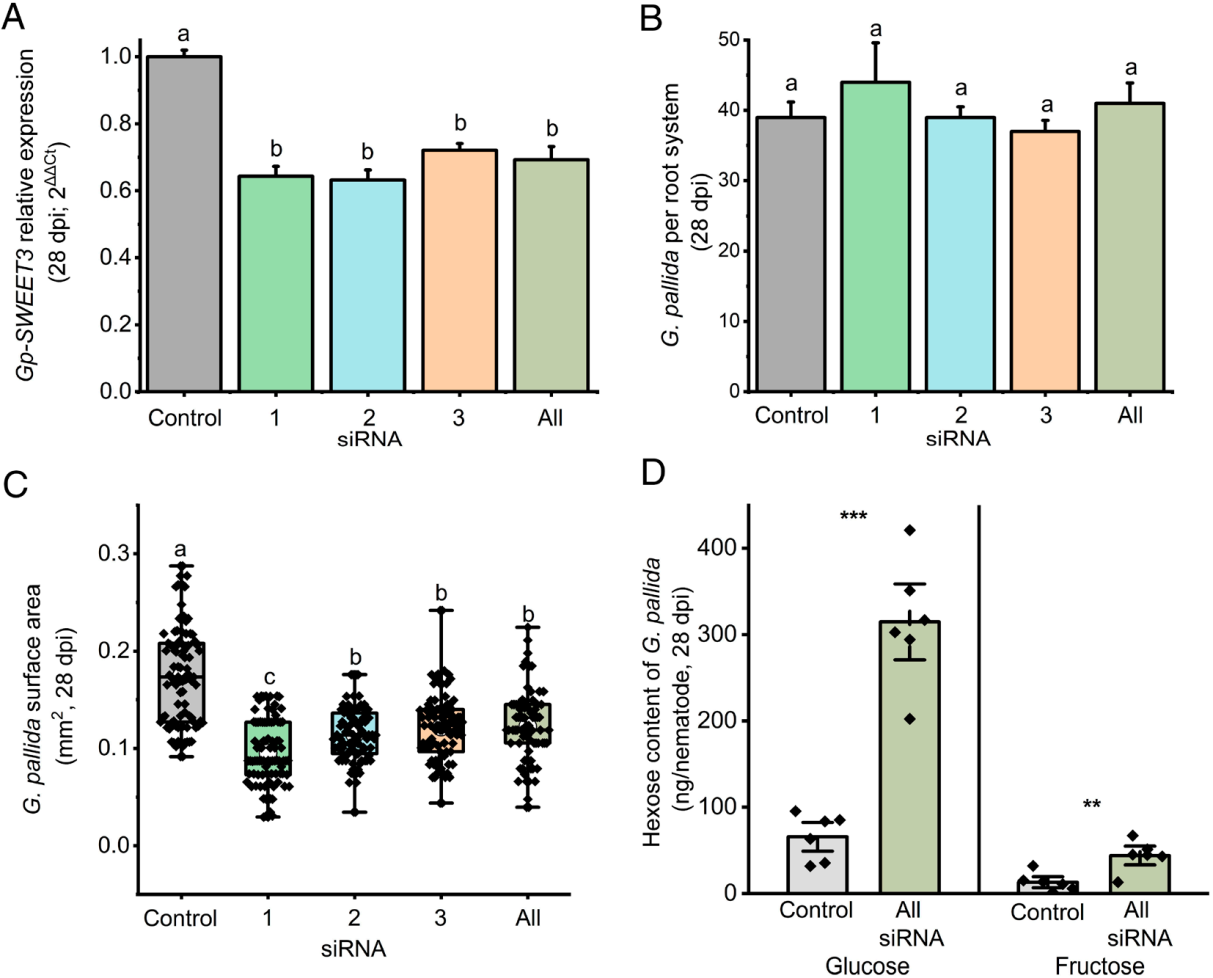
Knockdown of *Gp-SWEET3* reduces *G. pallida* growth and increases hexose concentrations. Three siRNA were designed that targeted three locations of the transcript and were incubated separately with second-stage nematode juveniles as well as one combined treatment (“All”), before inoculating onto potato roots. (*A*) qPCR was conducted on nematodes collected at 28 dpi to confirm the knockdown of *Gp-SWEET3* via RNAi. Elongation Factor was utilized as a reference gene for delta Ct analysis (28) relative to control nematodes treated with a scrambled siRNA with no *G. pallida* target. *G. pallida* were collected from roots by sieving to determine (*B*) the number of invaded nematodes and (*C*) their projected surface area. (*D*) The predicted substrates of *Gp-SWEET3*, glucose and fructose, were quantified in nematodes extracted from potato roots at 28 dpi via LC–MS. Letters denote significance at *P* < 0.05 (*A* and *B*) *P* < 0.01 (*C*) One way-ANOVA, SNK. Asterisks denote significance at *P* < 0.01 (**) and *P* < 0.001 (***) Two-sample *t* test (*D*). Six biological replicates were conducted for *A*–*D*. Error bars indicate SEM.

We repeated analyses at 28 dpi to determine whether reduced expression of *Gp-SWEET3* prematurely arrests development or results in a continued reduction in growth rate. At 28 dpi, there was a continued reduction in *Gp-SWEET3* expression (grand mean 35% reduction relative to control; Fig. 2*A*; *P* < 0.01; One way-ANOVA, SNK). Similar to 10 dpi (*SI Appendix*, Fig. S5*B*), reduced expression of *Gp-SWEET3* did not reduce total nematode numbers in the root (Fig. 2*B*); however, there was a marked reduction in nematode surface area (Fig. 2*C*; *P* < 0.01; One way-ANOVA, SNK). The surface area of *Gp-SWEET3*:siRNA-treated nematodes was on average 37% smaller than nematodes treated with the scrambled siRNA control, at 28 dpi. Knockdown of *Gp-SWEET3* resulted in an increase in the predicted substrates of SWEET3, glucose and fructose, within nematodes compared to controls (Fig. 2*D*; glucose *P* < 0.001, fructose *P* < 0.05; One way-ANOVA, SNK).

**Fig. 3.**
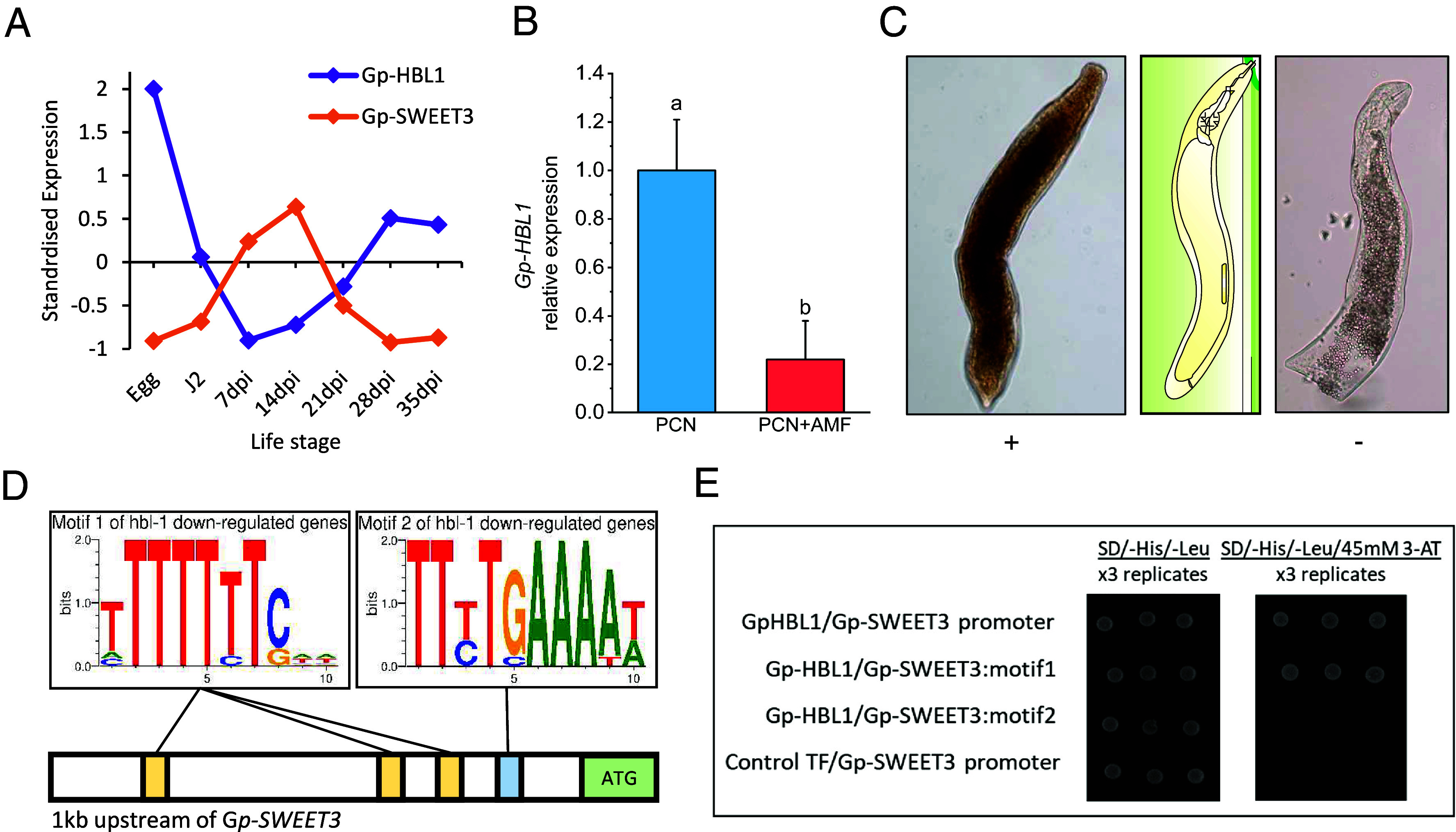
Gp-HBL1 negatively regulates *Gp-SWEET3* expression within the nematode intestine. (*A*) *Gp-HBL1* expression is negatively correlated with *Gp-SWEET3* expression throughout nematode development (Pearson’s r = −0.72, *P* value = 0.0015; z-score standardised data). (*B*) *Gp-HBL1* expression in *G. pallida* 5wpi in potato roots ± AM fungal colonization (*P* < 0.001; FDR DESeq2). (*C*) In situ hybridization of *Gp-HBL1* transcripts via digoxigenin probes on parasitic stages of *G. pallida*. Antisense probe (+) hybridization indicates gene expression localization, whereas a sense strand probe acts as a negative control and indicates background staining. (*D*) Presence of the predicted motif 1 and 2 from *C. elegans* genes negatively regulated by Ce-HBL1 (30), highlighted on the 1 kb stretch upstream of the *Gp-SWEET3* ATG. (*E*) Yeast-one hybrid assays confirmed an interaction between Gp-HBL1 protein and the *Gp-SWEET3* promoter, and motif 1 but not motif 2. A control DNA-interacting protein, FLO (Control TF), did not interact with the *Gp-SWEET3* promoter, supporting the specificity of the Gp-HBL1–*Gp-SWEET3* promoter interaction. Yeast growth on media lacking histidine, supplemented with 45 mM 3-AT (SD/-His/-Leu/45 mM 3-AT) is indicative of a protein–DNA interaction. Each test was performed in triplicate using independent transformants.

The *C. elegans* interactome (29) and our transcriptomic dataset suggested HBL1 as a likely transcription factor that regulates *SWEET3*. There was a significant negative correlation between the expression of *Gp-HBL1* (GPLIN_001077100) and *Gp-SWEET3* throughout the life cycle of *G. pallida* (Fig. 3*A*; Pearson’s r = −0.72, *P* = 0.0015), similar to that found in *C. elegans* studies (30). *Gp-HBL1* was down-regulated in nematodes collected from AM-colonized potato roots (Fig. 3*B*; −4.54-fold; FDR < 0.001). Similar to *Gp-SWEET3*, *Gp-HBL1* was expressed within the intestine of parasitic stages of *G. pallida* (Fig. 3*C* and *SI Appendix*, Fig. S6). Analysis of the *Gp-SWEET3* promoter sequence (1 kb upstream of the ORF) revealed an over-representation of HBL1 binding motif 1 and motif 2 (Fig. 3*D*; *P* < 0.001), which are also present in the promoters of the 53 *C. elegans* genes that are negatively regulated by HBL1 and similar to the A-enriched motifs in *Drosophila* HBL binding sites (30). *HBL1* was identified in all analyzed genomes of plant-parasitic nematodes (*SI Appendix*, Fig. S7).

We conducted yeast-one-hybrid assays to determine the interactions between Gp-HBL1 and potential binding motifs in the *Gp-SWEET3* promoter. The 1 kb promoter region, as well as three tandem copies of motif 1, interacted with Gp-HBL1, while motif 2 did not (Fig. 3*E*). The Antirrhinum flowering-related protein FLO (31) was used as a negative control and did not interact with any of the *Gp-SWEET3* promoter regions tested.

**Fig. 4.**
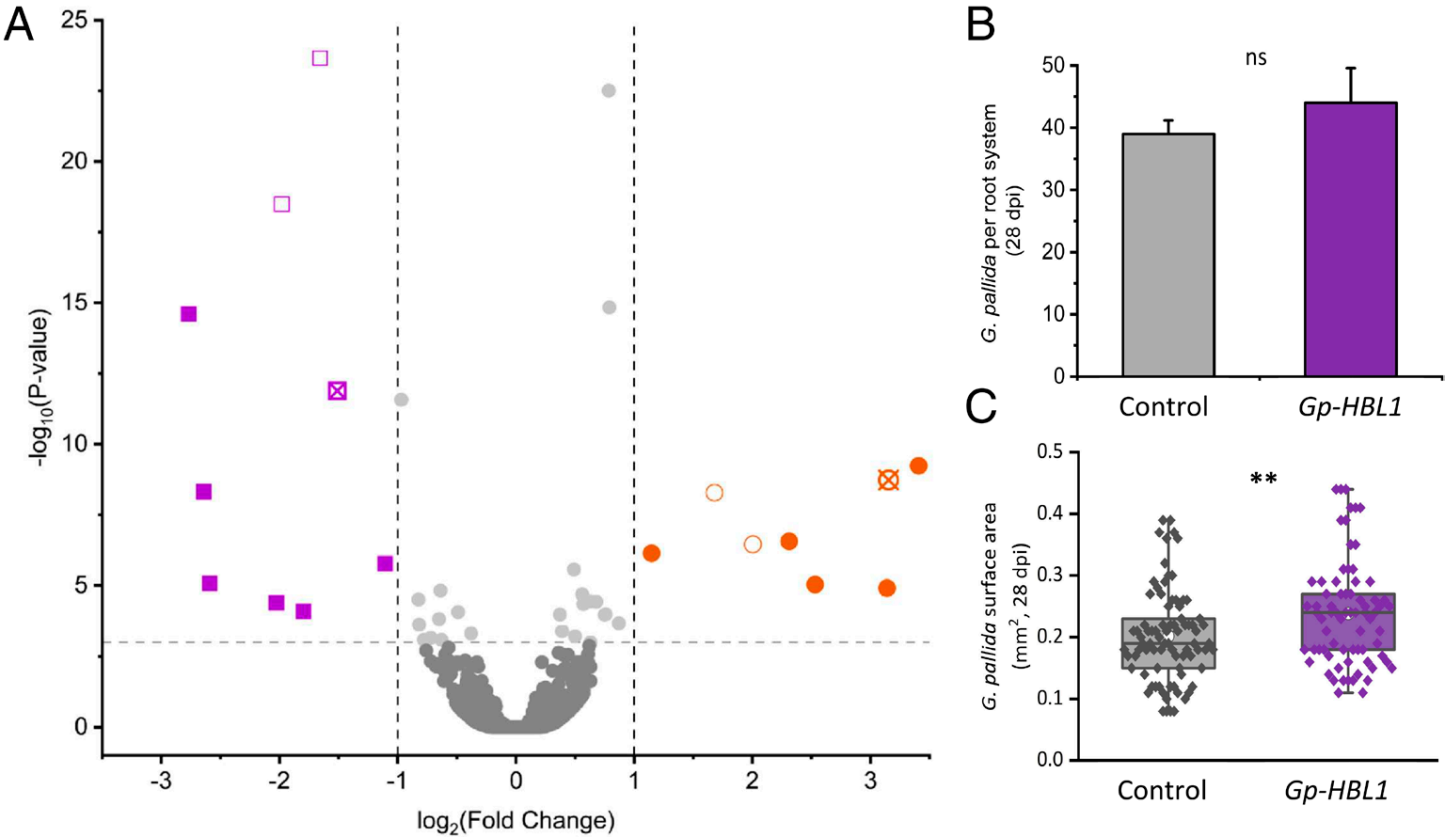
The impact of *Gp-HBL1* on nematode gene expression and parasitism. (*A*) *Gp-HBL1:*siRNA treated nematodes were inoculated onto potato roots and gene expression was assessed via RNA sequencing from nematodes collected at 28 dpi. Down-regulated (purple) and up-regulated genes (orange) are visualized by volcano plot, with triangles indicating genes with a promoter (1 kb upstream of ATG) containing the down-regulatory HBL1 binding motif. *Gp-HBL1* and *Gp-SWEET3* are shown by crossed-symbols in purple and orange, respectively. Scrambled, control siRNA with no homology to any nematode gene was used as a control treatment. (*B*) The number of siRNA-treated nematodes within potato roots at 28 dpi. (*C*) The surface area of siRNA-treated nematodes at 28 dpi (ImageJ). Six biological replicates were used for *A*–*C*. Statistical analysis was conducted through DESeq2 FDR < 0.001 1 < log_2_FoldChange < −1 (*A*); two-sample *t* test (*B* and **C) with asterisks denoting significance at *P* < 0.01 (**).

RNAi was conducted to investigate the interaction between *Gp-SWEET3* and the predicted negative regulator *Gp-HBL1*. Incubation of second-stage juveniles with three pooled *Gp-HBL1* targeting siRNAs resulted in a 51% reduction in Gp-HBL1 24 h posttreatment (*SI Appendix*, Fig. S8). In these same nematodes, there was a 7.6-fold increase in *Gp-SWEET3* expression (*SI Appendix*, Fig. S8), confirming a transcriptional effect on *Gp-SWEET3* from Gp-HBL1. *G. pallida* juveniles postknockdown of *Gp-HBL1* were then inoculated onto potato roots to determine long-term effects of RNAi and identify other genes that are potentially regulated by Gp-HBL1. There remained a significant impact of RNAi on *G. pallida* gene expression at 28 dpi (Fig. 4*A*; FDR < 0.001; 1 < log2 fold change < −1; *SI Appendix*, *Supplementary File* 2), confirmed by RNA-sequencing of collected nematodes, indicating long-term changes to gene expression profiles from exposure of juveniles to siRNAs. *Gp-HBL1* expression was reduced (−1.5 log2 fold change; Fig. 4*A*) while *Gp-SWEET3* expression was increased (+3.3 log2 fold change; Fig. 4*A*). Seven other *G. pallida* genes were upregulated in *Gp-HBL1* knockdown nematodes and represented lipase, fatty acid, and peptidase functions (*SI Appendix*, *Supplementary File* 2). The vast majority of nematode genes were unaffected by *Gp-HBL1* RNAi, despite 957 *G. pallida* genes containing the HBL1 motif 1 in their promoter regions. Promoter analysis suggests an enrichment of the motif 238 bp upstream of the start codon of these genes, with no strand specificity (*SI Appendix*, Fig. S9, Mean 237.7 SEM 2.5, Median 238). Seven of the eight genes negatively regulated by Gp-HBL1 (i.e., up-regulated in this study) were found to contain the predicted HBL1 binding motif, similar to *Gp-SWEET3* (Fig. 4*A*). Genes up-regulated by *Gp-HBL1* knockdown contained an average of 3.32 motifs in their promoter regions, compared to 1.24 motifs in *G. pallida* promoters that contain at least one motif, and 0.24 for the presence of the motif across all *G. pallida* promoter regions.

Knockdown of *Gp-HBL1* also resulted in reduced expression of nine other *G. pallida* genes (i.e., positive regulation). These genes represented lipase, nitrate transporter, fatty-acid binding, and secreted protein functions/predictions (*SI Appendix*, *Supplementary File* 2). Despite downstream expression impacts of *Gp-HBL1* knockdown, RNAi did not impact root invasion (Fig. 4*B*), however, treated nematodes were found to be significantly larger at 28 dpi (Fig. 4*C*) than control nematodes.

## Discussion

**Fig. 5.**
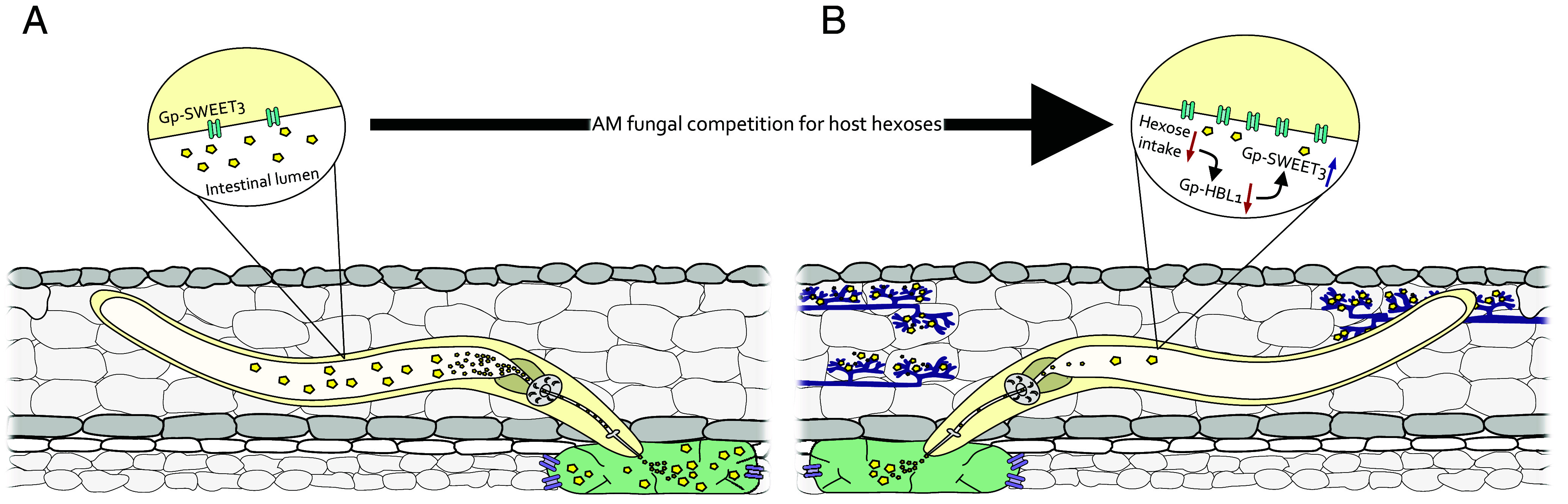
The role of SWEETs in host–nematode interactions. Plant-parasitic nematodes, such as the white potato cyst nematode *G. pallida*, induce the formation of complex, metabolically active feeding structures, termed syncytia (green), by inducing large-scale transcriptional changes within the vasculature of roots. This includes the induction of host plant SWEET genes (Sugars Will Eventually be ExporTed; transmembrane proteins; purple), which facilitate the influx of sugars (yellow) into the syncytium. (*A*) Hexose sugars are ingested by the feeding nematode from the syncytium and transferred across the intestinal lumen by nematode SWEETs (*Inset*), to fuel nematode development and reproduction. (*B*) AM fungi also rely upon host hexose supplies for their survival and may cocolonize nematode-infected root tissues. This competition results in a reduction in hexose intake by the parasitic nematode, which is perceived and leads to a down-regulation of the transcription factor gene *HBL1*. HBL1 represses *SWEET3* expression via conserved regulatory motifs and is coexpressed in the nematode intestine. Therefore, *HBL1* down-regulation results in the activation of *SWEET3*. We propose that derepression of *SWEET3* by HBL1 during hexose-limiting scenarios (e.g., during competition from AM fungi) is a mechanism to avoid the damaging effects of reduced hexose intake, by ensuring maximum utilization of available resources.

Ingestion of host resources is essential for the survival, development, and reproduction of obligate biotrophic plant parasites. Variations to this flux, either in quantity or quality, may have profound effects on parasite success and plant growth. These variations are inevitable when the parasites have to compete for the same range of host resources with other biotrophs, such as mutualistic AM fungi. Limiting the transfer of plant resources to biotrophs may have positive consequences for the plant (21). Our data show that when AM fungi colonize a host and induce systemic changes to host resource profiles (i.e., carbohydrate content), concurrent parasitic cyst nematodes (*G. pallida*) can perceive and respond to the consequential changes in their food intake. Specifically, we have shown that AM fungal colonization of the host plant reduces the hexose intake of concurrently infecting *G. pallida*. Within these nematodes, a putative diet-responsive transcription factor, *Gp-HBL1*, is down-regulated, which in turn leads to up-regulation of a hexose membrane transporter, *Gp-SWEET3*, in the intestine, presumably in an attempt to offset a perceived hexose deficit (Fig. 5). This negative regulation of *Gp-SWEET3* by Gp-HBL1 may be a mechanism by which a plant pathogen adjusts to dynamic host quality to optimize development and reproduction.

*G. pallida* infecting host root systems colonized by AM fungi receive a reduced supply of glucose and potentially fructose, compared to *G. pallida*-only roots, supporting previous studies that observed a reduction in total carbon flow to *G. pallida* in this system (15, 16). One explanation for this is competition between both symbionts for the same hexoses, which are known to be important for their development (3, 32–34). This would suggest systemic competition due to the localization of each symbiont in opposing halves of the split-root system and the presence of cyst nematodes within vascular tissues, whereas AM fungi colonize the root cortex (35). Systemic competition could be driven by the strength of their resource sinks (13). Additionally, as nematodes are individuals rather than potentially forming a united mass like AM fungi (36), intraspecies competition between individual nematodes may also play a role and reduce their fecundity.

The effect of AM fungi on pathogen molecular processes is relatively unknown compared to knowledge on their nutrition and priming effects on the host plant (35). Here, we found that distal AM-colonization induced differential expression of 582 nematode genes. These genes represent a range of nematode processes, with several key families suggested to be involved in cuticle/collagen modifications and resource modifications/transportation. Apparent changes to the nematode cuticle may be an indirect effect, associated with the nutritional supplementation of nematodes on AM-colonized roots altering their rate of development. This could shift the relative timing of the molts compared to nematodes on non-AM root systems resulting in differences in expression of cuticle-related genes. AM fungal colonization increases the fecundity of *G. pallida* females (15, 16, 37, 38), implying that changes to cuticle or collagen profiles may also enable the female to hold more eggs. Interestingly, genes related to lipid synthesis/modifications were both up- and down-regulated in nematodes from AM hosts. This may suggest that the lipid profile is also responsive to the intake of the nematodes at that particular time-point. Lipid composition is known to vary throughout the development of plant-parasitic nematodes (39), therefore, it is also potentially influenced by external factors such as plant–mutualist interactions that may affect the rate of development.

Here, we characterized the impact of AM fungi on *Gp-SWEET3*, a hexose transmembrane transporter that was up-regulated in nematodes receiving reduced glucose from their hosts. We propose that this gene is part of a feedback mechanism that is up-regulated to maximize the import and utilization of resources. A single SWEET gene is present in the genomes of most animals, with nematodes being the exception; *C. elegans* contains seven SWEET genes (40) and here we identified between 2 and 13 within plant-parasitic nematodes. *SWEET3* was conserved in all plant nematode genomes studied, including *P. coffeae*, which is the smallest plant nematode genome (26) with only two SWEET genes. *Gp-SWEET3* is expressed in the intestine and during feeding stages of *G. pallida*, therefore likely involved in feeding and utilizing ingested resources. Little is known about intestinally expressed genes of plant-parasitic nematodes due to a focus on secreted effectors (8, 9, 11, 41, 42), however they are essential for the utilization of plant compounds that then fuel their reproduction. Knockdown of *SWEET3* had no impact on the number of *G. pallida* within the roots at 10 and 28 dpi, however the nematodes were smaller and there was an increase in the concentration of glucose and fructose within these nematodes. This suggests that upon knockdown of *Gp-SWEET3* these hexoses become rate limiting for nematode growth and development if the nematode cannot remove them from their intestinal lumen, leading to their accumulation. Reduced growth may simply be due to their inability to utilize the compounds for downstream processes or due to toxic effects of high localized glucose concentrations that are known to have damaging effects on nematode lifespans due to increased formation of reactive oxygen species and mitochondrial dysfunction (43). This emphasizes the complex role of different nutrients in these damaging parasites and how they may adjust their molecular biology in response to positive plant–mutualist interactions to maximize their success. Nutrient flow into plant-parasitic cyst nematodes has been proposed to influence sex determination, with nematodes from nutritionally starved (including carbohydrate/polysaccharides) feeding sites differentiating into males (44). Our time points for analysis were selected for pre- and postexiting of males from the roots so that any impact on sex determination could be determined. As the same number of nematodes were observed in the roots at both time points relative to controls, this suggests that these hexoses are likely not the nutritional factors that may contribute to sex determination of *G. pallida*. After incubation of J2s in siRNA, we observed gene knockdown up to 28 dpi. RNAi has been found to be maintained across several plant-parasitic nematode generations (45, 46), suggesting that long-term knockdown can be achieved, as seen in our study. The expression peak of *Gp-SWEET3* later during nematode development may aid the long-term persistence and efficacy of siRNA within these nematodes.

In AM-colonized potato, we consistently observed greater fecundity of *G. pallida* despite a reduced influx of carbohydrates from their host (37, 38). This implies that nematode carbohydrate intake is not “rate-limiting” for egg production at the concentration as a result of concurrent plant–AM interactions, and other factors such as the elevated phosphorus and nitrogen quantities in these nematodes may play a greater role (15). It is possible that at higher colonization density of AM fungi than we observed, there may be greater competition for plant sugars, thereby limiting growth and egg production. In this scenario, the dynamic regulation of sugar transporters may be beneficial to maximize the utilization of the compounds the nematode ingests and reduce excreted quantities.

The identification of a diet-responsive gene suggests the presence of a diet-responsive regulator. To explore this, we analyzed differential gene expression from published reads (16) as well as searching the *C. elegans* “interactome” (29) for potential regulatory transcription factors. Combined approaches suggested that *Gp-HBL1* was down-regulated in nematodes that had elevated *Gp-SWEET3* expression and that Ce-HBL1 interacts with its *SWEET3* ortholog. Ce-SWEET3 is also predicted to interact with a noncoding RNA *rncs-1*, however, this does not appear to have an ortholog in plant-parasitic nematodes. HBL1 is a Cys-His zinc finger transcription factor that controls the transition from larvae to adults in *C. elegans* by inhibiting precocious gene expression. Heat-induced overexpression of *Ce-HBL1* results in the co-overexpression of 24 genes and the down-regulation of 53 genes via positive and negative regulatory mechanisms involving HBL1 and predicted promoter motifs of these genes (30). Studies in *Drosophila* and *C. elegans* have shown that *HBL1* orthologs are autonomously capable of either inducing or repressing different genes based on transcription factor concentrations, which are proposed to be regulated by the miRNA *let-7* (47, 48). Similar mechanisms may exist in cyst nematode species, which also contain *let-7* orthologs. Alternatively, while we observed a direct repressive interaction between Gp-HBL1 and *Gp-SWEET3*, HBL1 may activate genes indirectly through recruitment of other, as yet unknown, transcription factors via conserved promoter motifs among the genes induced by HBL1 (30).

Motifs similar to *C. elegans* HBL1 repressor motifs were found in the promoter of *Gp-SWEET3* and also enriched in other genes affected by *Gp-HBL1* knockdown. The regulatory promoter motif 1 was confirmed to interact with Gp-HBL1, indicating a diet-responsive factor that controls downstream diet-related genes. Initiation of feeding is a major step in the life cycle of *G. pallida* and feeding stages exhibit vast transcriptional responses (25). A lack of feeding will prevent progression to adult stages, therefore while we do not propose that Gp-HBL1 regulates development in the same way as for *C. elegans*, we suggest that this is due to their dissimilar life cycles. Preventing the precocious expression of feeding-related genes allows the parasite to devote more of its finite resources to establishing in the root system before it begins feeding and develops into an adult. *HBL1* is also present in all analyzed plant-parasitic nematodes, indicating that this diet-responsive feedback mechanism is perhaps conserved. The challenge is now to determine the primary mode of perception of food quality and how this initiates a molecular response, in the first instance. In *C. elegans*, the insulin signaling (IIS) pathway is suggested to respond to changes in glucose levels, inferring a role in IIS-regulated gene expression responses to dietary intake (49). The *G. pallida* genome contained orthologs of this evolutionarily conserved pathway, suggesting possible glucose-sensing mechanisms in plant-parasitic nematodes. *C. elegans* gustatory neuron activity is known to increase during low glucose concentrations (50), therefore, similar neuron-associated mechanisms may mediate hexose concentration-dependent gene responses in *G. pallida*.

We observed that a reduction in *Gp-SWEET3* expression was detrimental for nematode growth, while the overexpression of this gene via knockdown of *Gp-HBL1* yielded an increase in nematode size. This is perhaps due to increased SWEET3-mediated transportation of hexoses or the precocious expression of other HBL1-regulated genes that may result in earlier initiation of feeding. It is possible that the rate of HBL1-controlled growth may be at the maximum in this system. In these nematodes, there are almost certainly additional factors in their diet (e.g., nutrients) or additional growth-related genes that are now impeding development. Determining these additional factors and what is required to produce the optimal parasite will aid our attempts to limit these losses from the host and reduce the parasitic burden on agriculture. The transcriptomic analysis of *Gp-HBL1*:RNAi nematodes suggests that HBL1 controls the expression of other diet-related genes involved in metabolism and nutrient transport, therefore predicting other possible pathways that may respond to diet composition and control growth rates. Among these functions, lipid modifications were highly represented both up- and down-regulated, indicating complex changes to nematode lipids rather than merely the increase or decrease in their abundance. In *C. elegans*, nutrient-sensing pathways can be activated by lipase products and control metabolism and lifespan/development, inevitably regulating aging (51).

Overall, this study suggests that plant parasites are not acquiescent to external factors that may severely influence host resource abundance and composition, such as concurrent plant–mutualist interactions. Rather, they have an ability to discern shifts in their food intake and then respond via transcriptional mechanisms to attempt to balance deficiencies. This may be particularly important for cyst nematodes that exhibit a relatively long feeding period compared to other pathogens (4), therefore needing to persist through variations in host quality. We propose that this ability appears conserved throughout plant-parasitic nematodes based on gene conservation and targeting these “core” abilities of damaging parasites with diverse feeding mechanisms may assist control efforts.

## Materials and Methods

### Transcriptome Analysis.

Reads from Bell et al. (16) were obtained for RNA sequencing of potato roots split between *G. pallida* and AM fungal symbiosis, to investigate the impact of distal symbioses on each organism. Reads were mapped to a *G. pallida* genome (PRJNA702104) via HISAT2 (52). Gene counts were quantified by HTSeq (53) and analyzed by DESeq2 (53 to yield differential gene expression profiles of *G. pallida* parasitizing potato roots ± distal and concurrent AM fungal colonization. Differential expression cut-off filters were 1 < fold change < −1, and FDR *P* < 0.001. Differentially expressed genes with functions related to resource transport or synthesis, due to a differential resource profile of *G. pallida* in these roots (15), were filtered via gene ontology to indicate *Gp-SWEET3* as a gene of interest.

Orthologs of *Gp-SWEET3*, as well as the seven *C. elegans* SWEET genes, were identified in other major plant-parasitic nematode genomes by BLAST through WormBase Parasite (54). Amino acid sequences were obtained for the SWEET genes and aligned via CLUSTAL (55) before constructing a phylogenetic tree under maximum likelihood (56). The life stage expression of *SWEET3* from *M. incognita, H. schachtii,* and *G. pallida* were identified through published life stage transcriptome datasets (25, 57, 58), and the life stage (motile or feeding) at which the gene was expressed the highest was noted by pictogram on the phylogenetic tree.

### Growth of Plants and Cultures.

For pot experiments, single tubers of *Solanum tuberosum* cv. Désirée were planted in 23 cm diameter pots containing heat-sterilized sand:topsoil [50:50 RHS Silver Sand:Bailey’s Norfolk Topsoil, nutritional (37). To generate the *G. pallida* inoculum, plants were inoculated with *G. pallida* (population Lindley) and grown for 12 wk, until plant death, to enable reproduction. Roots were vigorously shaken in the soil to dislodge nematode cysts and yield a soil inoculum of *G. pallida* for future experiments. The *G. pallida* content of this stock soil was determined (59). For subsequent experiments, this stock soil was then mixed with sterilized sand:loam topsoil to get the desired *G. pallida* densities of 20 eggs per gram. AM fungal inoculum (*Rhizophagus irregularis*) was obtained from PlantWorks Limited, UK, in a zeolite and pumice carrier mixture. Pot soil was inoculated with 30 g of AM fungal inoculum placed directly under the tuber. Pots without AM fungi were mock-inoculated with carrier mix lacking AM fungal spores. Pots were randomized for layout in a controlled, containment glasshouse (18 °C/16 h day length, watered every other day).

To collect motile second-stage juvenile nematodes, cysts were collected from stock *G. pallida* soils (59) and the eggs were hatched in potato root diffusate. Diffusate was collected by soaking the roots of 3-wk-old potato plants in water (80 g root/L water) overnight. Hatched juvenile nematodes were then collected and stored at 10 °C.

### In Situ Hybridization.

Single-strand digoxygenin-labelled anti-sense DNA probes for *Gp-SWEET3* and *Gp-HBL1* were synthesized with DIG DNA labeling mix (Roche, Germany) from complementary DNA (cDNA) fragments amplified by primers *GPSWT3f*: ACCGTGTGGTCCTTCTTCAG, *GPSWT3r*: CTCAAGCTATGCATCCAACG, *GPHBLf*: TGATGCAAGAACAGCCAGAC, *GPHBLr*: GCGTCTTTGAAGGCAATCTC. Sense probe controls were constructed in separate reactions as negative controls. These probes were used for in situ hybridization to determine spatial expression patterns for both genes. Two cm sections of potato roots 3 wk post inoculation with *G. pallida* were fixed in 10% formaldehyde for 48 h at 22 °C. Roots were then blended and fixed nematodes were extracted via sieves. Fixed nematodes were individually hand-cut with a razor blade before washing with M9 buffer and proteinase-K treatment (2 mg/mL for 90 min at 22 °C). The method was then continued as described previously (28). Specifically, nematodes were frozen and treated with methanol for 1 min followed by acetone for 1 min before rehydration in RNase-free water. Treated nematodes were hybridized with the probes overnight at 50 °C and then washed three times with 4× saline sodium citrate (SSC) and three times with 0.1× SSC/0.1% SDS at 50 °C. Nematodes were incubated at 22 °C in 1% blocking reagent in maleic acid buffer (Roche, Germany) for 30 min and labeled for 2 h with anti-digoxigenin-AP Fab fragments 1:1,000 in 1% blocking reagent. The nematodes were stained overnight at 4 °C with 337 µg/mL nitroblue tetrazolium and 175 µg/mL 5-bromo-4-chloro-3-indolyl phosphate. Stained nematodes were washed in 0.01% Tween-20 before viewing and imaging.

For hybridization chain reactions, nematodes were collected and fixed as above before following in situ HCR protocols (60) using HCR probes produced by Molecular Instruments Inc., USA.

### Gene Knockdown via RNAi.

27mer dicer-substrate small interfering RNAs were designed for *Gp-SWEET3* and *Gp-HBL1* using the design tool supplied by Integrated DNA Technologies available at https://eu.idtdna.com/pages/products/functional-genomics/dsirnas-andtrifecta-rnai-kits. Each sequence was checked to ensure no off-target effects, via BLAST. An off-target match was designated as a sequence that shares >16 base identity to the first 19 bases of the siRNA.

Six pools of approximately 4,000 *G. pallida* J2s were incubated at room temperature in 100 mM octopamine and either target gene siRNA (20 µg) or negative control siRNA (20 µg) that constituted a scrambled sequence with no target in *G. pallida*. For *Gp-SWEET3*, three siRNA were designed that targeted three locations of the transcript and were incubated separately as well as one combined treatment. Nematodes were incubated for 24 h, 72 h, 7 d, or 10 d to observe the impact of incubation length. For *Gp-HBL1,* there was a single treatment that consisted of all three siRNAs, due to the lack of additive effects observed for *Gp-SWEET3* RNAi. Nematodes were incubated for 24 h due to lack of extended incubation for *Gp-SWEET3* knockdown. For either RNAi, the nematodes were then washed five times in tap water. Approximately 500 nematodes were removed for infection assays while the remaining were frozen for RNA extraction.

Total RNA was prepared from nematode samples using an E.Z.N.A Plant RNA Kit according to the manufacturer’s protocol including DNase treatment (Omega Biotek, UK). First-strand cDNA was synthesized from 750 ng RNA using iSCRIPT (BioRad, UK) following the manufacturer’s protocol. Analysis of gene expression was carried out using qRT-PCR with Brilliant III Ultra-Fast SYBR® Green Master Mix (Agilent Technologies, CA). Cycle conditions were 95 °C for 30 s and subsequently 40 cycles of 5 s at 95 °C and 10 s at 60 °C. Primer pairs *GPSWT3* and *GPHBL* detailed above had amplification efficiencies of 95 and 97%, respectively. The 2^−ΔΔCt^ method was used to calculate relative expression between control and experimental samples with six biological and three technical replicates (61).

To assess the wider effects of Gp-HBL1 RNAi, collected RNA was sequenced (Azenta, USA). Reads were analyzed as described above to yield differentially expressed genes in Gp-HBL1 knockdown nematodes. The sequencing data can be found under NCBI BioProject PRJNA1167637.

One hundred of the treated nematodes were infected onto roots of 7-d-old potato plants grown in soil-free pouches, as previously described (62). The 100 nematodes were distributed between up to five root tips on the root system. Each treatment was replicated six times. Plants were grown in water with a weekly addition of general-purpose fertilizer. Nematodes were then collected by blending the roots and extracting by sieves, as mentioned above. The surface area of approximately 20 nematodes from each replicate was quantified by ImageJ (63).

### HILIC Amide LC–MS of Glucose and Fructose.

Approximately 50 nematodes were collected per root system, as described above, and subsequently stored at −80 °C before subjected to a 30 min ultrasound-assisted extraction, using ethyl acetate and methanol (35:65 v/v) at room temperature. The samples were centrifuged at 3,500 rpm for 3 min. Supernatants were freeze-dried and reconstituted in 100 µL 90 AcN/5% water, vortexed for 30 s, sonicated for 2 min, and centrifuged at 10,000 rpm for 20 min.

Five µL of each sample or the glucose and fructose standard mixture in 90% AcN was injected into a Vanquish LC system (ThermoFisher Scientific, UK) using a flow rate of 0.25 mL min^−1^. The analytical column was Accucore-150-Amide-Hydrophilic Interaction Liquid Chromatography (HILIC) (2.6 um particle size, 150 mm × 2.1 mm, ThermoFisher Scientific, UK) held at 35 °C. Starting mobile phase composition was 90% solvent B (0.1% ammonium acetate in acetonitrile) in A (0.1% ammonium acetate and water) decreasing to 60% B over 5 min and held constant for 4 min before being re-equilibrated to 90% B after 2 min. Column eluant was eluted in to an Orbitrap Exploris 240 mass spectrometer (ThermoFisher Scientific, UK) and ionized using electrospray ionization in negative polarity at 2,500 V. Mass measurement used full scan mode resolution of 120,000, a m/z range of 50 to 500. The maximum injection time was set automatically by the software. Samples and standards were analyzed in triplicate and Tracefinder 5.1 (ThermoFisher Scientific, UK) was used to construct the calibration curve and determine peak areas of hexose signals in the samples. Calibration standard concentrations analyzed were 1 ng, 10 ng, 100 ng, 1 µg, and 10 µg.

### Yeast One-Hybrid.

Yeast one-hybrid assays were conducted to test for transcription factor–promoter interactions by MATCHMAKER One-Hybrid System (Clontech, UK). Bait constructs were prepared by cloning the 1 kb region upstream of the *Gp-SWEET3* coding region or three tandem repeats of motif 1 or motif 2 into the pHisi vector. Bait constructs were transformed into yeast (YM4271) with the “Quick & Easy Yeast Transformation Mix” (Takara, US). The Gp-HBL1 prey construct was prepared by cloning the *Gp-HBL1* CDS in-frame with the GAL4 activation domain in the pGADT7-Rec2 plasmid. As a negative control prey, we used pGADT7-Rec2-FLO (31). Prey constructs were transformed into the various *Gp-SWEET3* bait yeast strains as above. Yeast were grown on SD/-Leu/-Trp (as a positive growth control condition) and SD/-His/-Leu medium supplemented with 45 mM 3-amino-1,2,4-triazole (3-AT), for assessing interactions. For each test, three independent transformant yeast colonies were selected and grown to an OD600 of 0.6. Five μL aliquots of each culture were spotted onto the appropriate SD plates and incubated at 30 °C for 3 d before imaging. All imaged colonies were derived from the same plate.

### Promoter Analysis.

The 1 kb upstream region of *Gp-SWEET3* was analyzed for potential transcription factor binding sites. Specifically, the identified binding motifs within the 53 promoters of genes that are negatively regulated by Ce-HBL1 (30) were located within the *Gp-SWEET3* promoter. The *GP-SWEET3* promoter was run-through MEME analysis (64) alongside the 53 *C. elegans* promoters to confirm the occurrence of a conserved HBL1 binding motif among all predicted nematode binding targets.

The prevalence of the identified motif within the genome was determined by analyzing the 1,000 bp upstream regions of every *G. pallida* gene. Upstream regions, termed promoter regions, were obtained from the *G. pallida* genome using get_upstream_regions.py (https://github.com/peterthorpe5 GNU GENERAL PUBLIC LICENSE). The incidence of the motif within these promoter regions was identified using the ‘Find Individual Motif Occurrences’ (FIMO) web server (65). These data were then applied to the *Gp-HBL1* RNAi analysis to indicate which up/down-regulated genes contain the proposed regulatory motifs.

### Data Visualization.

Plots were generated, and statistical tests were conducted, with OriginPro (66). Phylogenetic trees were generated by IQ-TREE (56).

## Supplementary Material

Appendix 01 (PDF)

Dataset S01 (XLSX)

Dataset S02 (XLSX)

## Data Availability

RNA sequencing data have been deposited in SRA (PRJNA1167637) (67).
