# Supplementary material for: Diet-regulated transcriptional plasticity of plant parasites in plant–mutualist environments
Source: Proc Natl Acad Sci U S A. 2025 Apr 17;122(16):e2421367122. doi: 10.1073/pnas.2421367122 (PMC12037023; doi:10.1073/pnas.2421367122)
Supplement: Supplementary file 1 — Appendix 01 (PDF) [file pnas.2421367122.sapp.pdf]

PNAS

Supporting Information for

Diet-regulated transcriptional plasticity of plant parasites in plant-mutualist environments

M Willow H Maxwell[1], Barry E Causier[1], Jasper Chippendale[1], James R Ault [2], Christopher A Bell[1*].
[1] School of Biology, Faculty of Biological Sciences, University of Leeds, Leeds, LS2 9JT, United Kingdom
[2] School of Molecular and Cellular Biology, Faculty of Biological Sciences, University of Leeds, Leeds, LS2 9JT, United Kingdom

*Corresponding author
Email: c.a.bell@leeds.ac.uk

This PDF file includes:

Figs S1 to S10
Tables S1 to S3
Legends for Datasets S1 & S2.
SI References

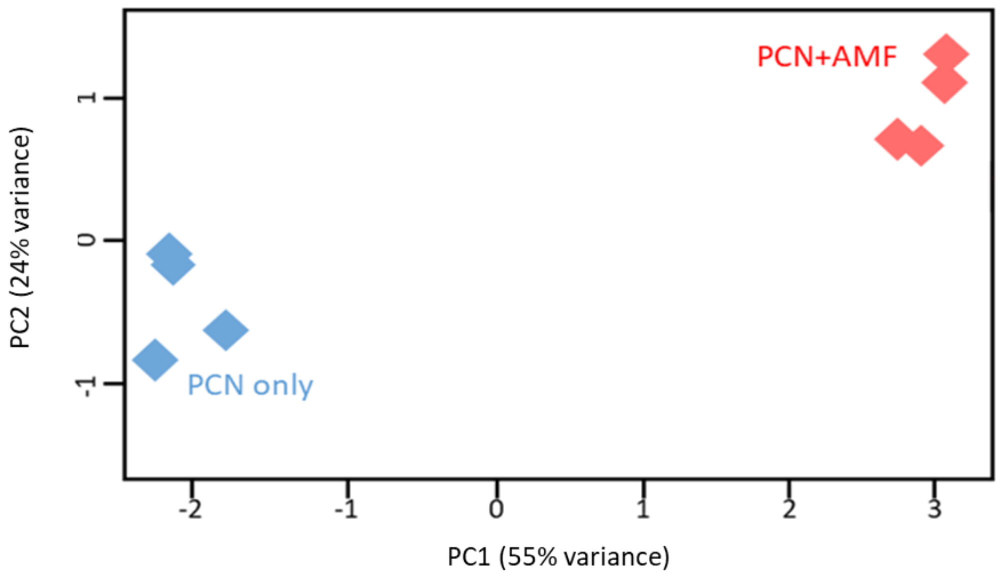

Fig S1: Plant-mutualist interactions induce transcriptional responses in root parasites. Distal colonisation of potato roots by AM fungi indirectly affects the gene expression profile of infective *G. pallida* at 5wpi. DESeq2 PCA of gene counts.

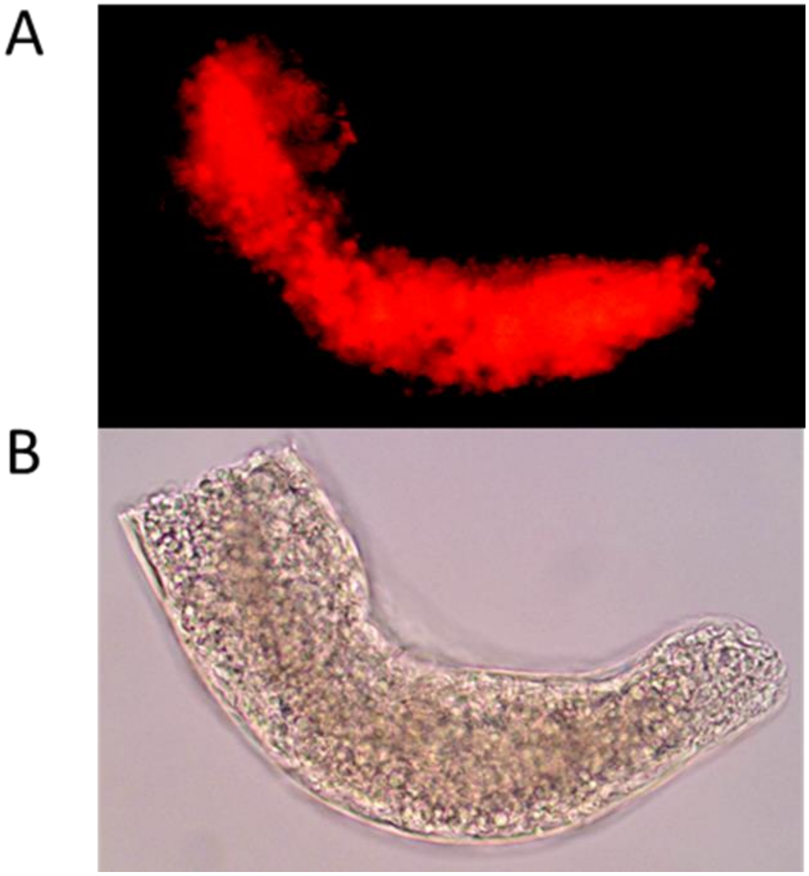

Fig S2: *In situ* hybridisation chain reaction of *Gp-SWEET3*. Parasitic J4 stages of *G. pallida* were probed by HCR to determine the spatial expression of *Gp-SWEET3*. Fluorescent (A; 647nm) and bright field (B) imaging.

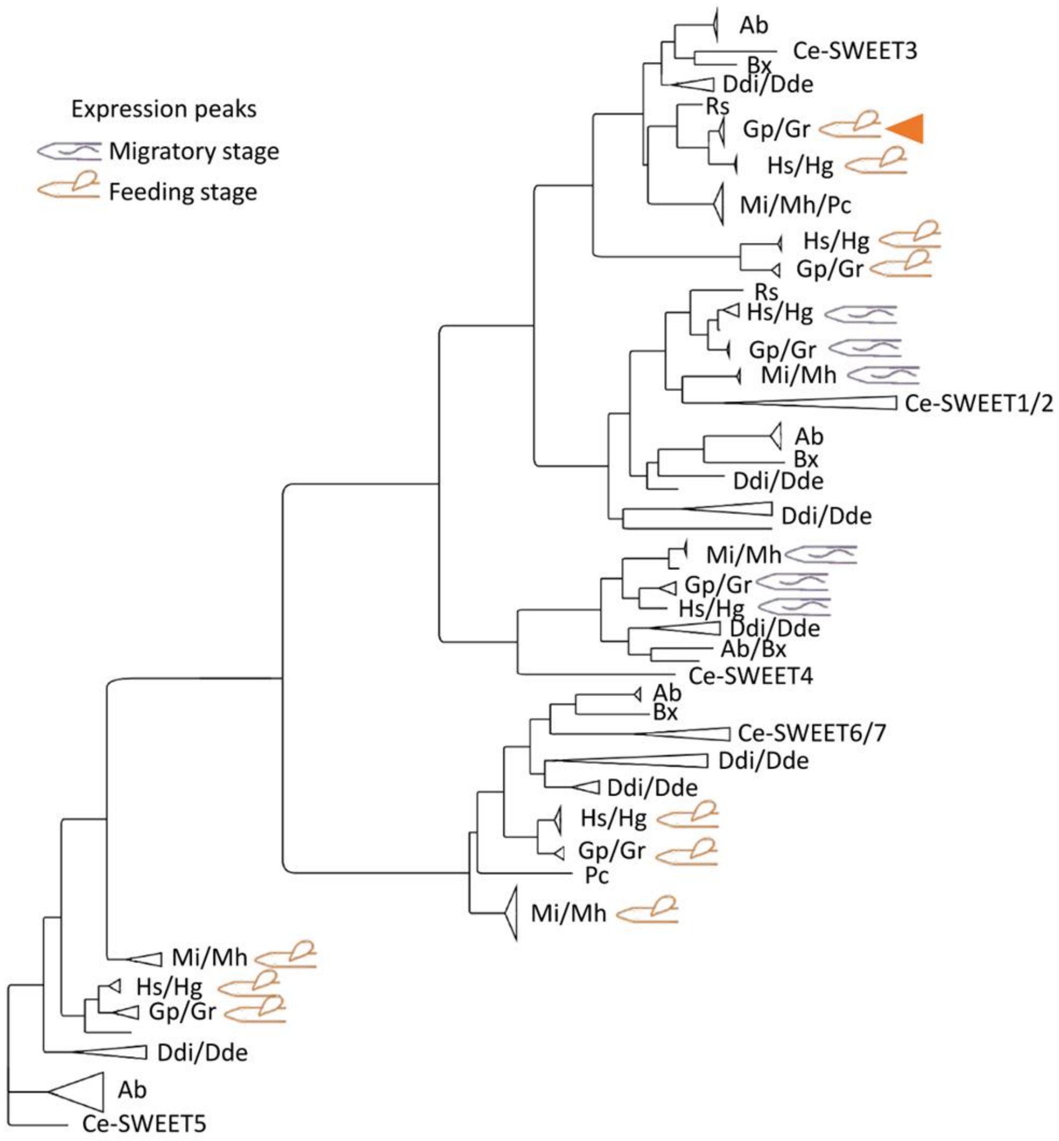

Fig S3: The maximum likelihood phylogeny of SWEET genes from plant-parasitic nematodes. *C. elegans* SWEET genes were BLAST searched against the genomes of diverse plant-parasitic nematodes; *Globodera pallida* (Gp), *Globodera rostochiensis* (Gr), *Heterodera schachtii* (Hs), *Heterodera glycines* (Hg), *Meloidogyne incognita* (Mi), *Meloidogyne hapla* (Mh), *Ditylenchus dipsaci* (Ddi), *Ditylenchus destructor* (Dde), *Radopholus similis* (Rs), *Pratylenchus coffeae* (Pc), *Aphelenchoides besseyi* (Ab), *Bursaphelenchus xylophilus* (Bx). Pictogram key indicates if the gene is expressed most highly in feeding or motile life stages of species with available life stage transcriptome data. Triangular node end size indicates an increasing number of grouped genes. Arrowed gene is *Gp-SWEET3*.

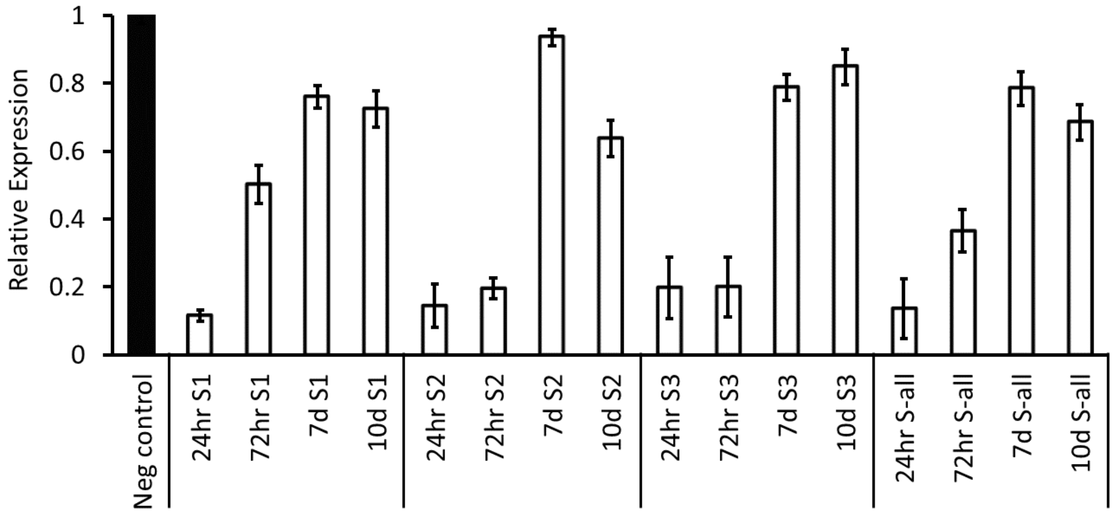

Fig S4: Expression of *Gp-SWEET3* in *G. pallida* J2s. 500 second-stage juveniles were exposed to siRNA for 16 hours, washed three times in tap water and then incubated in tap water at 20°C for 24 hr, 72 hr, 7 d or 10 d before quantification of *Gp-SWEET3* expression via qPCR. Three siRNA were designed that targeted three locations of the transcript and were incubated separately as well as one combined treatment ("All"). Expression is displayed relative to nematodes incubated in scrambled control siRNA (no *G. pallida* target) followed by incubation in tap water for the corresponding period. Elongation Factor was utilised as a reference gene for delta Ct analysis (1). Error bars indicate standard error of the mean for six pools of nematodes per treatment.

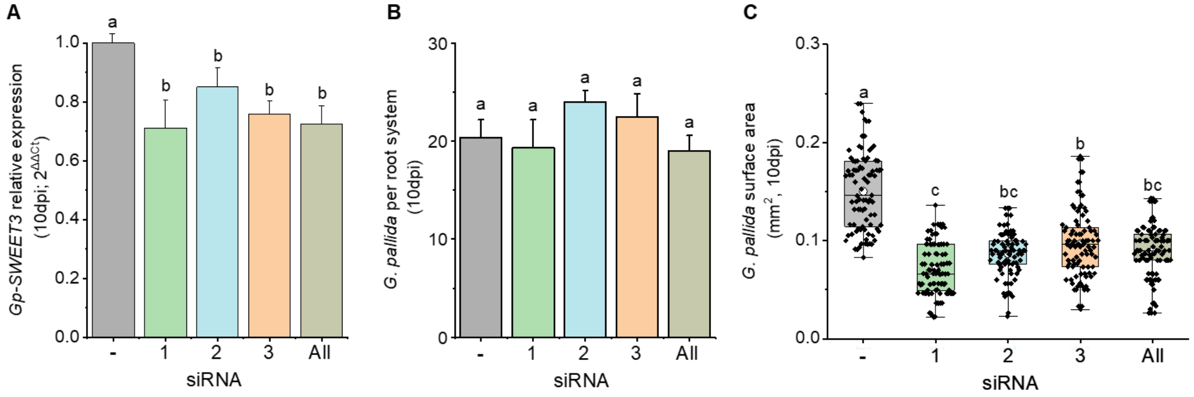

Fig S5: Knockdown of *Gp-SWEET3* reduces nematode growth but not root invasion of *G. pallida* at 10 dpi. A) qPCR was conducted on nematodes collected at 10 dpi to confirm knockdown of *Gp-SWEET3* via RNA-interference. Elongation Factor was utilised as a reference gene for delta Ct analysis (1), relative to control nematodes treated with a scrambled siRNA with no *G. pallida* target. Three siRNA were designed that targeted three locations of the transcript and were incubated separately as well as one combined treatment ("All"). B) The number and C) surface area projection of nematodes within potato roots at 10 dpi. Six biological replicates were conducted for A, B & C.

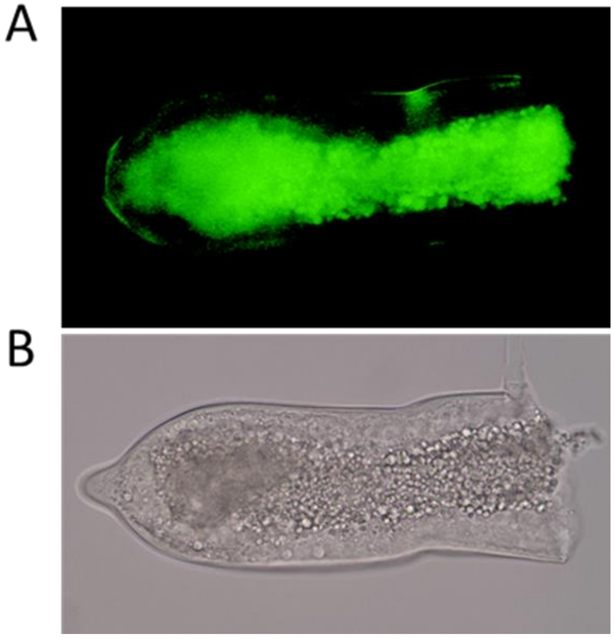

Fig S6: *In situ* hybridisation chain reaction of *Gp-HBL1*. Parasitic J4 stages of *G. pallida* were probed by HCR to determine the spatial expression of *Gp-HBL1*. Fluorescent (A; 488nm) and brightfield (B) imaging.

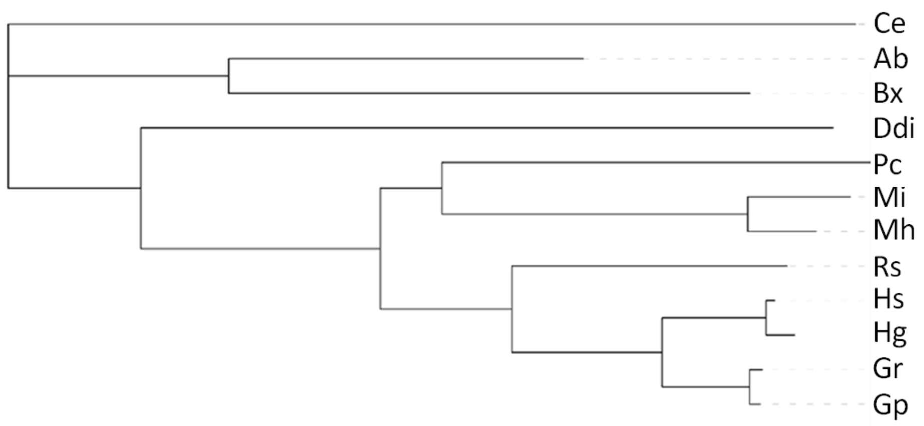

Fig S7: The maximum likelihood phylogeny of *HBL1* in plant-parasitic nematodes. *Gp-HBL1* was BLAST searched against the genomes of *Caenorhabditis elegans* (Ce; outgroup) and diverse plant-parasitic nematodes; *Globodera rostochiensis* (Gr), *Heterodera schachtii* (Hs), *Heterodera glycines* (Hg), *Meloidogyne incognita* (Mi), *Meloidogyne hapla* (Mh), *Ditylenchus dipsaci* (Ddi), *Radopholus similis* (Rs), *Pratylenchus coffeae* (Pc), *Aphelenchoides besseyi* (Ab), *Bursaphelenchus xylophilus* (Bx).

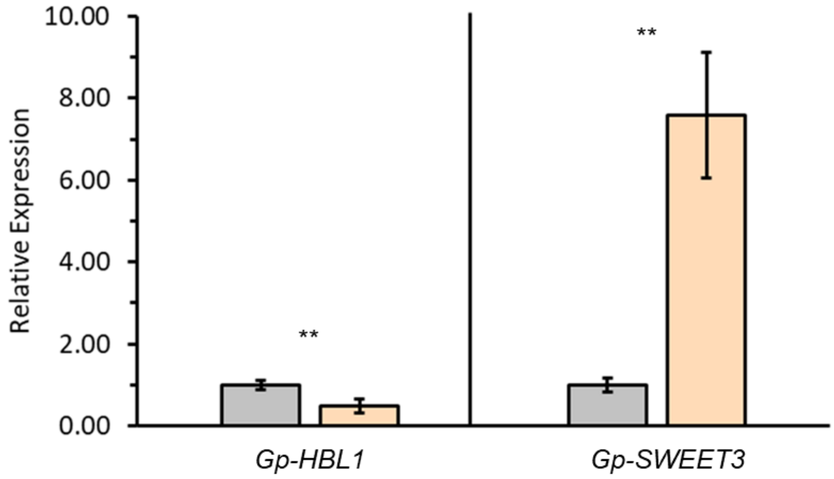

Fig S8: Knockdown of *Gp-HBL1* by RNAi increases the expression of *Gp-SWEET3.* siRNAs were synthesised and incubated with second-stage juveniles of *G. pallida* for 24 hours, then washed multiple times in water before qPCR analysis of both *Gp-HBL1* and *Gp-SWEET3*. siRNA treatment consisted of a combined solution of three independent siRNA. Elongation Factor was utilised as a reference gene for delta Ct analysis (1), relative to control nematodes treated with a scrambled siRNA with no *G. pallida* target. Four biological replicates were used, each containing 1000 *G. pallida* nematodes. Asterisk denote significance; P<0.01 Two-sample t-test.

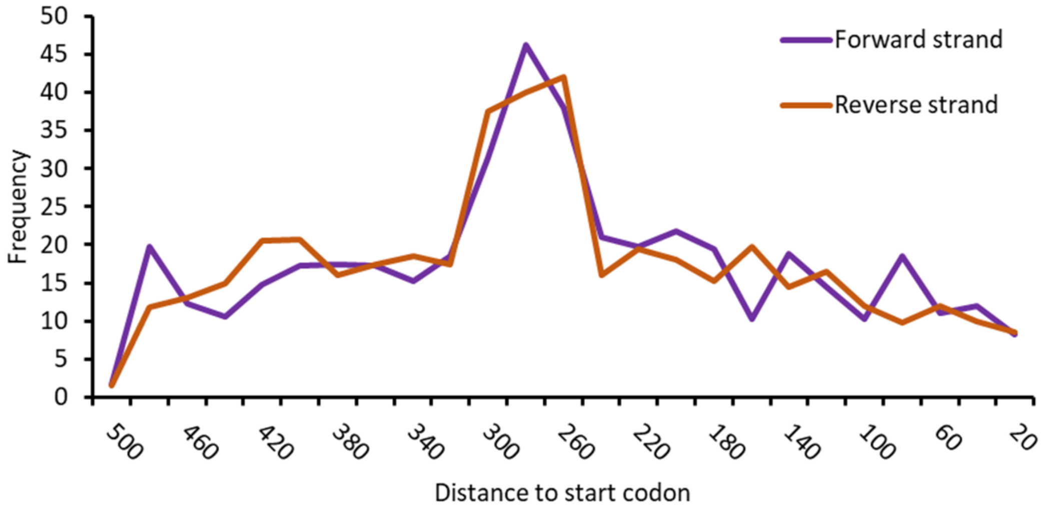

Fig S9: Frequency of Gp-HBL1 binding motif 1 across 500 bp of motif-containing promoter regions. Promoter regions of *G. pallida* genes were analysed via FIMO to identify 957 HBL-1 motif 1-containing promoters. The start sites of the motif throughout these promoters were analysed for motif start site frequency.

Table S1: Alignment of split-root potato RNA sequencing to *G. pallida* genome assembly. Reads were aligned to a *G. pallida* reference genome (SA_Gpal_Newton) to obtain the impact of distal AM fungal colonisation on nematode gene expression.

Treatment	Total reads in *G. pallida* root section	Percentage of reads aligned to *G. pallida* (Newton)
Gpal/control	48164367	8.3
Gpal/control	48285110	9.4
Gpal/control	30684150	9.3
Gpal/control	43754115	8.8
Gpal/AM fungi	40606208	8.2
Gpal/AM fungi	41879262	19.9
Gpal/AM fungi	48610258	8.1
Gpal/AM fungi	44197799	15.9

Table S2: The number of SWEET genes in a diverse range of plant-parasitic nematode species. Genes were identified as orthologues to annotated *C. elegans* SWEET genes via BLAST searches.

Nematode	# of SWEET genes
Caenorhabditis elegans	7
Globodera pallida	7
Globodera rostochiensis	6
Heterodera schachtii	6
Heterodera glycines	7
Meloidogyne incognita	13
Meloidogyne hapla	2
Radopholus similis	3
Pratylenchus coffeae	2
Aphelenchoides besseyi	12
Bursaphelenchus xylophilus	5
Ditylenchus destructor	7
Ditylenchus dipsaci	8

Table S3: Alignment of RNA sequencing reads from *Gp-HBL1* siRNA-treated nematodes to *G. pallida* genome. *G. pallida* juveniles were treated with siRNA and then inoculated onto potato roots. Negative control treatments included a scrambled siRNA that had no known match to any *G. pallida* gene. Three siRNAs targeting *Gp-HBL1* were pooled in the single HBL treatment. Each treatment had six replicates. At 28 dpi the nematodes were handpicked from the roots and RNA was purified for sequencing. Reads were aligned to the *G. pallida* reference genome to determine the impact of *Gp-HBL1* RNA interference.

		Reads aligned to *G. pallida* (Newton)	
Treatment	Total reads	Percentage	Number
Negative 1	34,027,241	80.15	27,272,834
Negative 2	33,044,310	85.80	28,352,018
Negative 3	46,418,978	87.09	40,426,288
Negative 4	46,023,422	87.28	40,169,243
Negative 5	49,782,989	86.45	43,037,394
Negative 6	48,751,011	84.36	41,126,353
HBL 1	34,969,892	84.49	29,546,062
HBL 2	37,705,362	85.45	32,219,232
HBL 3	30,947,271	81.28	25,153,942
HBL 4	31,357,135	83.40	26,151,851
HBL 5	32,658,347	82.34	26,890,883
HBL 6	33,536,814	88.36	29,633,129

Supplementary File 1: Differentially expressed genes in *Globodera pallida* infecting potato roots that are co-colonised by arbuscular mycorrhizal (AM) fungi, compared to non-AM colonised controls. Genes are filtered by FDR < 0.001, 1<log2 fold change<-1. File contains GO term annotation for each gene.

Supplementary File 2: Differentially expressed genes in *Globodera pallida* infecting potato roots post-knockdown of the transcription actor gene *HBL1.* Genes are filtered by FDR < 0.001, 1<log2 fold change<-1. Gene expression was quantified at 28 dpi.

SI References

1. S.C. Taylor, et al, The ultimate qPCR experiment: producing publication quality, reproducible data the first time. Trends Biotech. 37, 761–774 (2019).
